# Adjunctive Hyperbaric Oxygen Therapy in the Multimodal Management of Refractory Gluteal Hidradenitis Suppurativa: A Case Report

**DOI:** 10.1155/cris/6205080

**Published:** 2026-04-08

**Authors:** Arthur Cury Féres, Gabriel Valentim Souza, Rogério Serafim Parra, José Joaquim Ribeiro da Rocha, Omar Féres, Marley Ribeiro Feitosa

**Affiliations:** ^1^ Ribeirão Preto Medical School-USP, Av. Bandeirantes, 3900, Ribeirão Preto-SP, CEP 14049-900, Brazil; ^2^ Department of Surgery and Anatomy, Ribeirão Preto Medical School-USP, Av. Bandeirantes, 3900, Ribeirão Preto-SP, CEP 14049-900, Brazil

**Keywords:** hidradenitis suppurativa, hyperbaric oxygenation, multimodal therapy, skin transplantation, surgical flaps

## Abstract

**Background:**

Hidradenitis suppurativa (HS) is a chronic, inflammatory skin disorder characterized by recurrent nodules, abscesses, and sinus tracts, frequently leading to significant morbidity. Severe cases often require surgical intervention, and multimodal approaches are increasingly being explored to optimize outcomes.

**Case Presentation:**

We report the case of a 49‐year‐old male with long‐standing, treatment‐refractory Hurley III HS affecting the gluteal and proximal thigh regions. Despite lifestyle modifications and pharmacologic therapies, including antibiotics, methotrexate, and biologic treatment with adalimumab, there was no sustained clinical improvement. The patient subsequently underwent wide local excision followed by perioperative adjunctive hyperbaric oxygen therapy (HBOT) and reconstructive skin grafting as part of a multimodal salvage strategy. First, a wide local excision of the affected regions was performed, targeting areas with the most extensive disease activity. In the second stage, HBOT was administered to enhance granulation, reduce edema, and promote wound bed preparation. Finally, the patient underwent plastic reconstruction with split‐thickness skin grafts and a tie‐over dressing. Postoperative recovery was uneventful, with excellent graft integration and significant improvement in local inflammation, pain, and functional status. The patient was discharged ambulating and resumed daily activities without significant limitations.

**Conclusion:**

This case highlights the potential role of HBOT as a perioperative adjunct in refractory gluteal HS after failure of anti‐TNF therapy with adalimumab. In this setting, the combination of wide surgical excision, adjunctive HBOT, and reconstructive skin grafting resulted in satisfactory healing and functional recovery.

## 1. Introduction

Hidradenitis suppurativa (HS) is a chronic skin disorder characterized by inflammation and fibrosis, with a global prevalence ranging from 0.1% to 4.0% and more commonly diagnosed in young adults (third and fourth decades of life) [1, 2]. HS etiology involves factors that are not completely understood, such as genetic predisposition, immuno‐hormonal disturbances, exposure to environmental triggers and microbiological changes [3, 4]. Autoimmune dysregulation appears to play a central role toward hyperkeratosis, follicular hyperplasia, and occlusion [4]. The rupture of the pilosebaceous unit with subsequent release of keratin and bacteria into the dermis is a stimulus for chronic and progressive inflammation observed in most patients [5].

The hallmarks of HS include the development of deep‐seated painful nodules, abscesses, and draining sinus tracts, frequently accompanied by malodorous purulent discharge and progressive fibrosis/scarring. The disease affects predominantly areas that are rich in apocrine glands, such as the axillae, inguinal regions, and inframammary folds [5]. Notably, the involvement of the perineal and gluteal regions is particularly debilitating, often resulting in reduced quality of life, functional impairment, chronic pain, and psychosocial distress [6]. In fact, the prevalence of depression among HS patients ranges from 1.6% to 42.9% [7].

The management of HS should be individualized and progressive, tailored according to the clinical stage of the disease. Lifestyle modification remains a cornerstone across all stages, including stress reduction, wearing breathable clothing, maintaining local hygiene, hair removal in affected areas, smoking cessation, moderation of alcohol intake, dietary adjustments based on individual triggers, and weight loss when indicated [8].

Smoking and obesity are considered potential contributing factors in the development of the disease and are also associated with a poorer prognosis. Evidence suggests that nicotine may stimulate abnormal glandular secretion and disrupt neutrophil chemotaxis, thereby contributing to increased disease severity [9, 10].

Several studies have demonstrated a strong association between smoking and perianal HS, with smoking reported in up to 70% of affected patients. Therefore, smoking cessation is regarded as a key component of disease management [10–12]. Obesity may exacerbate HS by promoting sweat retention and contributing to mechanical disruption of follicular and glandular openings, thereby facilitating inflammation and lesion development [11].

Topical and/or systemic antibiotics are typically recommended for mild‐to‐moderate forms of HS. In moderate‐to‐severe disease, anti‐TNF biologic therapy, particularly with adalimumab, has demonstrated efficacy in reducing chronic inflammation and improving patients’ quality of life [13, 14].

Surgical intervention is often indicated in cases of therapeutic failure or when complications arise. Commonly employed techniques include: incision and drainage, deroofing procedures with or without marsupialization, and localized or wide excisions, with or without reconstruction using grafts or flaps. For patients considered candidates for surgery, multidisciplinary follow‐up is essential to ensure optimal outcomes [15, 16].

Hyperbaric oxygen therapy (HBOT) has emerged as a valuable adjunct to surgical treatment, especially in cases requiring extensive excisions or complex reconstructions. By enhancing tissue oxygenation and reducing the risk of infectious complications, HBOT may accelerate wound healing and improve graft integration in reconstructive procedures [17, 18].

This case report aims to describe the clinical course of a patient with extensive, treatment‐refractory HS of the gluteal region who underwent a multimodal approach involving wide excision, bridging therapy with hyperbaric oxygen, and subsequent reconstruction with a split‐thickness skin graft.

## 2. Case Report

A 49‐year‐old male patient presented with a longstanding HS, initially diagnosed at the age of 20. He had no known comorbidities aside from his underlying dermatological condition. Since the initial diagnosis, the patient had undergone multiple therapeutic regimens without sustained clinical improvement. Previous treatments included nonpharmacological measures, namely smoking cessation (achieved 5 years before admission), weight reduction with body mass index improvement from 31 to 27 kg/m^2^, local hygiene measures, and dietary counseling aimed at caloric restriction and healthier food choices, with weight loss achieved without the use of GLP‐1 receptor agonists, as well as pharmacological therapy with topical clindamycin, oral doxycycline, combination oral therapy with clindamycin and rifampicin, and systemic immunomodulation with methotrexate. Despite these interventions, the disease remained persistently active. In the 2 years preceding hospital admission, the patient had been maintained on biological therapy with adalimumab at a standard maintenance dose (40 mg/week, subcutaneously). Nevertheless, there was no significant clinical response, and the patient continued to exhibit ongoing inflammatory activity with extensive areas of involvement. Other biologic options, including secukinumab, were not used because, although approved in Brazil for HS, they were not available through the Brazilian Unified Health System for this indication at the time of treatment. In view of the persistent refractory gluteal disease, the patient was therefore referred to surgical evaluation.

**Figure 1:**
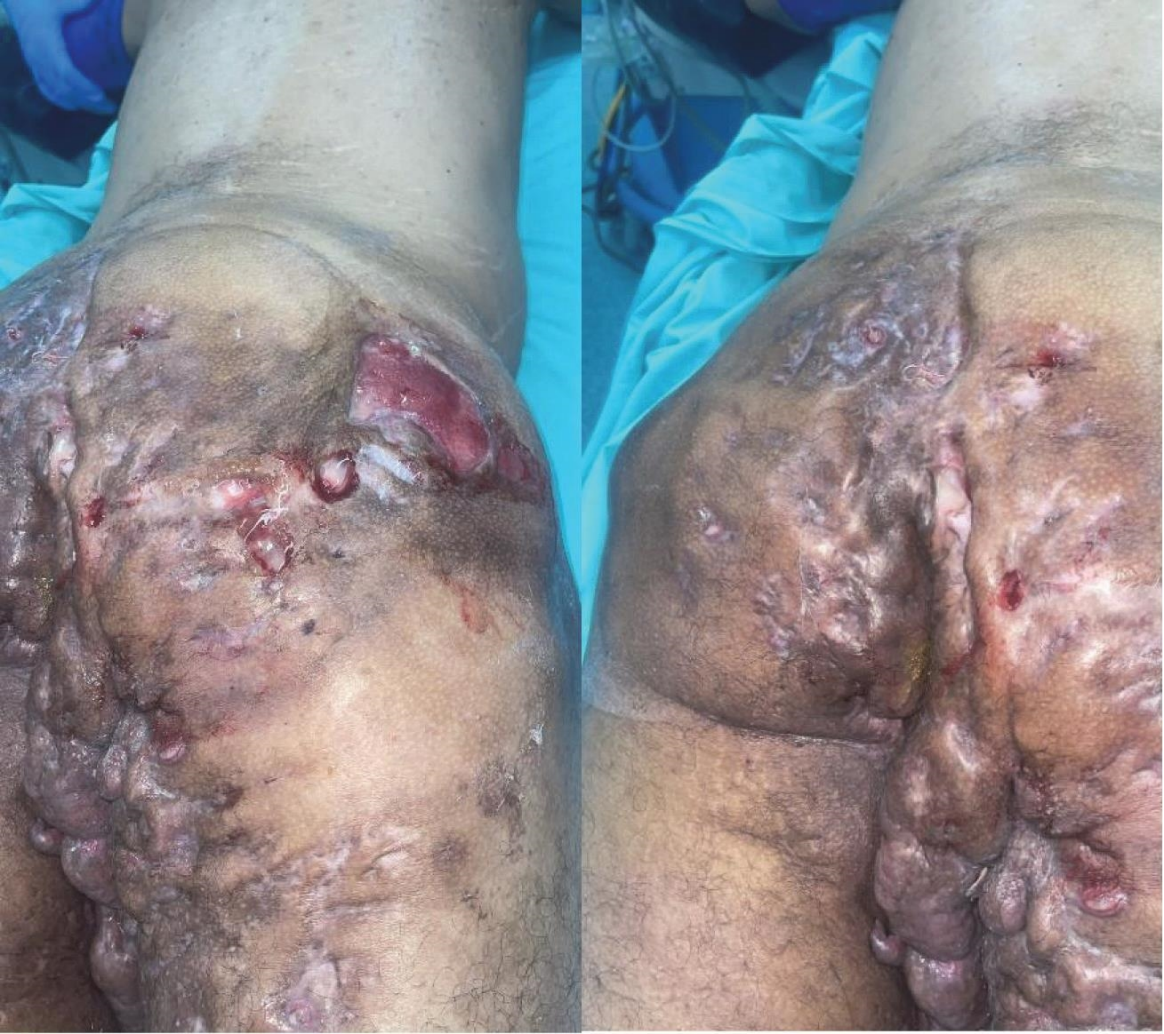
Advanced‐stage hidradenitis suppurativa lesions observed in the gluteal region and proximal thighs.

On physical examination, advanced‐stage HS lesions were observed (Hurley stage III). In the gluteal region and proximal thighs, there were multiple deep subcutaneous nodules and malodorous purulent discharge. The lesions were accompanied by areas of induration, surrounding erythema, local warmth, and tenderness to palpation. The affected tissue formed a bridged pattern of chronic inflammation, significantly impairing the patient’s ability to carry out daily activities and affecting his social interactions (Figure 1). Laboratory evaluation revealed mild leukocytosis (12,300/mm^3^) with neutrophilic predominance (78%). C‐reactive protein (CRP) was elevated at 21.6 mg/L (reference range: <5 mg/L). Hemoglobin level was 13.9 g/dL, platelet count was 310,000/mm^3^, and liver and renal function tests were within normal limits. Coagulation profile, serum glucose, and electrolytes were also within reference ranges.

**Figure 2:**
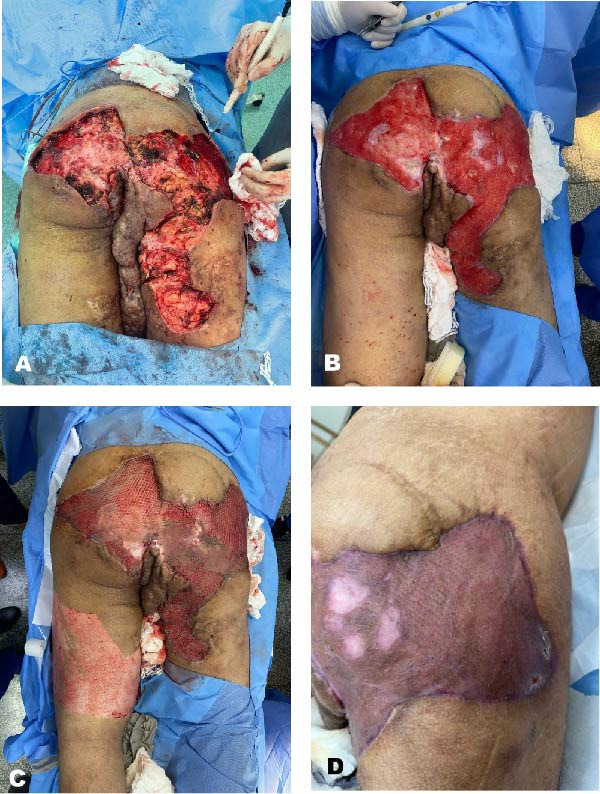
Multimodal treatment strategy in managing advanced hidradenitis suppurativa. (A) Immediate appearance after wide excision of the areas with active disease. (B) Satisfactory healing with exuberant granulation tissue after 12 sessions of hyperbaric oxygen therapy. (C) Immediate appearance after the application of split‐thickness skin grafts. (D) Excellent integration of the skin grafts, 2 weeks after plastic reconstruction.

Given the failure of optimized medical management and the chronic, refractory nature of the condition, the patient was referred for multimodal surgical therapy, conducted in three stages. In the first stage, a wide excision of the areas with the highest disease activity (gluteal region and the right proximal thigh) was performed. The procedure was uneventful, with no intraoperative complications or need for blood transfusions. On the first postoperative day, the second stage was initiated, consisting of HBOT. A total of 13 consecutive sessions were carried out in a monoplace chamber (2800 Sechrist Monoplace Hyperbaric Chamber, Sechrist, USA), pressurized to 2.4 atmospheres absolute (ATA). Each session lasted 2 h. Following this period, the patient demonstrated good wound granulation and a significant reduction in edema, with no signs of local infection. The final stage involved a partial‐thickness skin graft to cover the extensive raw area, particularly in the perineal region, combining the grafting technique with the application of a tie‐over dressing at the donor site, located on the left thigh. After 14 days following the plastic surgery procedure, the patient exhibited favorable graft take and satisfactory tissue recovery, allowing for hospital discharge with resumed ambulation. The patient has remained under follow‐up for 12 months, with sustained good disease control and no recurrence of lesions during this period (Figure 2).

## 3. Discussion

Treating HS remains a significant challenge, largely due to the incomplete understanding of its underlying causes and the frequent lack of response to conventional therapies, particularly in more advanced stages of the disease. In general, treatment should follow a stepwise, individualized approach guided by the severity and extent of the disease. In clinical practice, the Hurley staging system or the International HS Severity Score System (IHS4) may be used to assess disease progression and plan treatment strategies [5].

In early‐stage disease, conservative measures, such as lifestyle modifications, topical clindamycin, and, in some cases, short courses of oral antibiotics like tetracyclines, are often effective in controlling symptoms. In moderate cases, systemic antibiotics combined with immunosuppressive agents may be required to manage flare‐ups and limit progression. For more severe and treatment‐resistant forms, biologic therapy targeting anti‐TNF (adalimumab) and IL‐17 (secukinumab and bimekizumab) has emerged as a promising and effective option for disease control [5, 17]. In the present case, other biologic options, including secukinumab, were not used because, although approved in Brazil for HS, they were not available through the Brazilian Unified Health System for this indication at the time of treatment.

Despite the advances in medical therapy, a subset of patients fails to respond to pharmacologic treatment and requires surgical intervention. In the present case, the main novelty lies not merely in the use of HBOT in HS but in its application as a perioperative adjunct after failure of anti‐TNF therapy with adalimumab in a patient with refractory gluteal disease requiring extensive surgical and reconstructive management. Wide local excision remains the definitive therapy for extensive HS, with recurrence rates significantly lower than those associated with incision and drainage or deroofing techniques. However, excisional surgery is often associated with delayed wound healing, risk of infection, and functional impairment, necessitating a comprehensive, multimodal treatment strategy, as proposed [16].

In this context, surgical resection was combined with adjunctive HBOT, which may be particularly useful in optimizing the wound bed and supporting postoperative healing in complex reconstructive settings [17, 19]. HBOT enhances tissue oxygenation, promotes angiogenesis, and facilitates neovascularization, thereby improving the quality of the wound bed and preparing it to receive skin grafts more effectively. Additionally, HBOT contributes to the reduction of perilesional edema, modulation of inflammatory cytokines, and suppression of local infection, creating a more favorable environment for wound healing and graft integration [20, 21].

HBOT may contribute to postoperative recovery not only by increasing tissue oxygen tension, but also by modulating biological pathways involved in inflammation control and tissue repair. Elevated levels of reactive oxygen species (ROS) are associated with enhanced pathogen clearance [22]. Furthermore, ROS stimulates the production of several growth factors, including vascular endothelial growth factor (VEGF), placental growth factor (PGF), and angiopoietins 1 and 2 (Ang‐1 and Ang‐2), and promotes the recruitment of bone marrow‐derived stem cells, which play a key role in neovascularization [23]. Taken together, these effects suggest that HBOT acts through coordinated vascular, reparative, and immunomodulatory mechanisms, rather than through oxygen delivery alone; this interpretation is further supported by human studies showing modulation of inflammatory gene‐expression programs under chronic wound conditions [22–24].

The use of split‐thickness skin grafts following wide excision of HS lesions plays a key role in restoring skin integrity. By reducing the duration of open wound exposure, secondary complications such as infection, fibrosis, or chronic drainage may be minimized. Reconstructive techniques, whether through grafts or flap coverage, are essential not only to accelerate postoperative recovery but also to improve both functional and cosmetic outcomes, ultimately helping patients return more quickly to their daily routines [25].

This case underscores the importance of a tailored, multimodal treatment strategy in managing advanced HS, and, although evidence remains limited, HBOT may represent a useful perioperative strategy in selected patients with refractory gluteal HS following biologic treatment failure. The integration of surgical excision, HBOT, and reconstructive techniques offers a synergistic approach that may enhance healing, minimize complications, and improve short‐term outcomes in patients with treatment‐refractory disease. However, despite the encouraging results observed, further studies are needed to determine whether the long‐term benefits of multimodal treatment are sustained and whether they are associated with improved quality of life in this subset of the HS population.

## 4. Conclusion

In selected cases of refractory gluteal HS, particularly after failure of anti‐TNF therapy with adalimumab, HBOT may serve as a useful perioperative adjunct to extensive surgical excision and reconstructive treatment. In the present case, this multimodal strategy was associated with favorable healing and functional recovery.

## Acknowledgments

An AI‐based language assistance tool was used exclusively for English language editing.

## Funding

The authors received no financial support for the research, authorship, and/or publication of this article.

## Disclosure

The authors were fully responsible for the scientific content, clinical interpretation, and final revision of the manuscript. The funding received does not influence the study’s design, data collection, analysis, interpretation, or the writing of the manuscript.

## Consent

The participant has provided written informed consent for the submission of the case report to the journal.

## Conflicts of Interest

The authors declare no conflicts of interest.

## Data Availability

The data that support the findings of this study are available upon request from the corresponding author. The data are not publicly available due to privacy or ethical restrictions.
